# Case of IV Stage Juvenile Nasopharyngeal Angiofibroma Presurgically Treated with a Single ECA Stop-Flow Embolization Technique Using Onyx 18

**DOI:** 10.1155/2023/1351982

**Published:** 2023-05-04

**Authors:** Eliodoro Faiella, Domiziana Santucci, Davide Fior, Federica Riva, Chiara Tagliaferri, Laura Demelas, Giovanni D' Aniello, Rosa Maria Muraca, Maurizio Bignami, Lorenzo Paolo Moramarco

**Affiliations:** ^1^Department of Radiology, Sant' Anna Hospital, Via Ravona 22042, San Fermo Della Battaglia, Como, Italy; ^2^Unit of Computer Systems and Bioinformatics, Department of Engineering, Campus Bio-Medico University, Via Alvaro Del Portillo 21- 00128, Rome, Italy; ^3^Medicine and Surgery Faculty, Agostino Gemelli University Polyclinic, IRCCS, Via Della Pineta Sacchetti 217, Rome, Italy; ^4^Department of Otolaryngology, Sant'Anna Hospital, Via Ravona 22042, San Fermo Della Battaglia, Como, Italy

## Abstract

**Background:**

Juvenile nasopharyngeal angiofibroma (JNA) is a rare tumor that mainly affects young boys. Its intervention may be complex due to its high vascularity, location, and extension. Preoperative embolization is used to prevent intrasurgical and postsurgical bleeding. Two main kinds of embolization are described in literature: intratumoral and transarterial, and numerous embolic materials are used. *Case Presentation*. We want to present a case of presurgical embolization of a stage IV JNA, performed using a single stop-flow balloon assisted technique with the balloon cuffed exclusively in the external carotid artery and using Onyx 18 as an embolic agent.

**Conclusions:**

The embolization with an exclusive external carotid artery single stop-flow technique using Onyx 18 is a safe, effective, and a definitive approach.

## 1. Background

Juvenile nasopharyngeal angiofibroma (JNA) is a rare entity, comprising about 0.05% of all tumors of the head and the neck [1–3], found primarily in young boys. It is histologically considered a highly vascularized benign tumor, originating in the sphenopalatine region, but it is characterized by aggressive behavior with a tendency for rapid growth and invasion of neighboring structures, as well as by recurrence after intervention [4]. JNA tends to present with nasal obstruction and frequent episodes of relapsing epistaxis [2]. Surgical removal represents the most appropriate therapeutic approach [1]. In the recent years, endoscopic removal techniques have shown excellent results in terms of safety and efficacy also for large and infiltrative JNA, with lower complications compared to traditional open procedures, in terms of blood loss, hospital stays, and postsurgery recovery times [5, 6]. Presurgical vascular embolization (PVE) of the tumor lesion, especially in the head and neck areas, is now a well-established technique. It has the aim of reducing intraoperative blood loss and controlling intractable tumor bleeding [7, 8], and it is performed 24–48 hours prior to the resection. The traditional embolization technique is the transarterial approach by catheterization utilizing various embolic agents, such as microparticles, ethylene-vinyl alcohol copolymer [9], coils, or glue. Intratumoral embolization is a valid and a more recent technique.

We want to describe a case of a IV stage JNA (Fisch staging scale) [2] in a young boy, endoscopically removed and previously treated by transarterial embolization with a balloon-assisted technique cuffed in the external carotid artery using Onyx 18 as an embolic agent.

## 2. Case Presentation

A 19-year-old boy (C.M.) presented at the interventional radiology department of our hospital for presurgical embolization of the IV stage juvenile nasopharyngeal angiofibroma, using the Fisch staging scale [2]. He presented unilateral nasal respiratory obstruction and right epistaxis from 6 months. On the external previous CT, a voluminous right nasosinusal neoformation with an expansive growth, 60 × 30 × 55 mm, was detected in the nasal cavity, involving the ethmoid, sphenoid sinus, and medial pterygoid muscle. The skull base appeared remodeled, with erosion of the anterior portion of the great wing of the sphenoid, of the superior portion of the pterygoid process medial lamina, and of part of the right maxillary sinus medial wall. In addition, erosion of the sphenoid body at the level of the clinoid tract of the ICA was reported. A diagnosis of the IV stage of juvenile nasopharyngeal angiofibroma was suspected.

At the previous ENT evaluation, the endonasal endoscopy showed septal deviation with left anteroposterior crest from mass effect, and in the right nasal cavity, a voluminous neoformation completely occupied the nasal cavity, pulsated, and covered with whitish fibrin, fragile and easily bleeding.

The stadiation of the pathology was performed with contrast medium-MRI examination.

**Figure 1:**
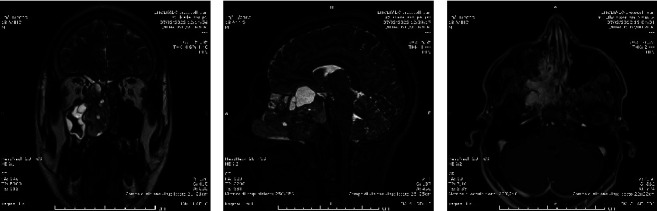
Preprocedural MRI scan: coronal (a) and sagittal (b) T2-weighted views showing large JNA of right nasopharyngeal cavity with expansion of the pterygopalatine fossa and extension into the infratemporal fossa with solid and cystic component. (c) Gd-enhanced T1 gradient-echo 3D axial image showed early and strong diffuse enhancement of the lesion.

The MRI reported a large mixed lesion, mainly solid and with some intralesional cystic portions, characterized by an intermediate signal in all sequences with numerous vascular flow voids in context. The lesion occupied the right nasal cavity projecting into the obliterated nasopharyngeal lumen. Medially, it imprinted the nasal septum. Laterally, it occupied the pterygopalatine fossa and the medial portion of the infratemporal fossa with remodeling and lysis of the bone profiles and interruption of the middle cranial fossa and portion adhering to the dura. Cranially, the lesion extended to the posterior ethmoidal region with remodeling of the floor of the anterior cranial fossa and imprinting on the optic chiasm, on the adjacent vascular structures, and on the pituitary gland and its pedicle, towards the right cavernous sinus (Figure 1). The lesion showed early enhancement and was supplied by branches of the bilateral internal maxillary artery with right dominancy. A diagnosis of JNA was made, and indication to hospitalization for surgery was therefore given.

Before surgical procedure, an embolization was required.

**Figure 2:**
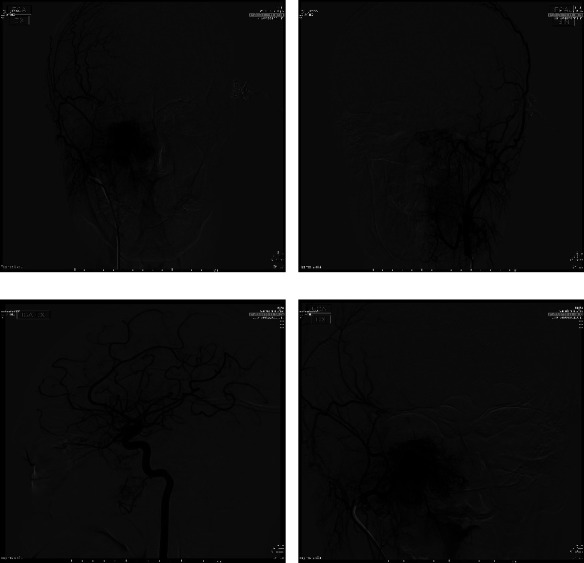
Preoperative DSA: selective catheterization of proximal right ECA and ICA. Posteroanterior (PA) (a–c) and detailed (d) views of the JNA with major feeders from sphenopalatine branches of the distal right internal maxillary artery. Some small feeders also arise from right ICA branches (c). Controlateral ICA did not show any feeders.

**Figure 3:**
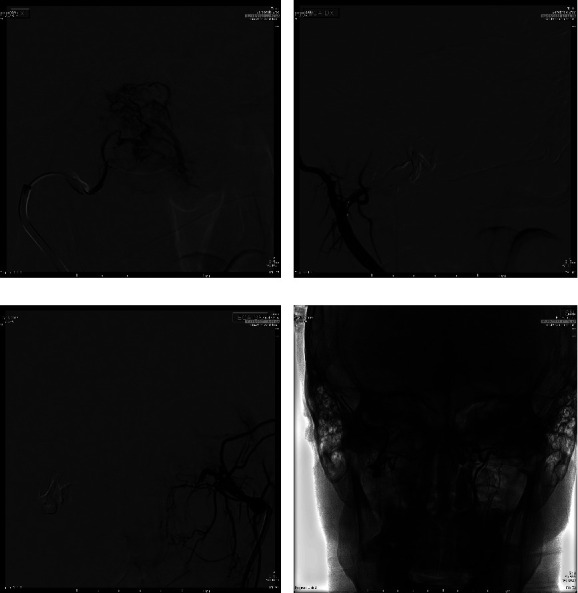
(a) Intraprocedural DSA: PA view of superselective injection of distal internal maxillary artery with onyx embolization. (b, c) Postprocedural DSA: LL view of the JNA showing successful embolization of the lesion of right (b) and left (c) side. (d) Postprocedural fluoroscopy demonstrating excellent results.

**Figure 4:**
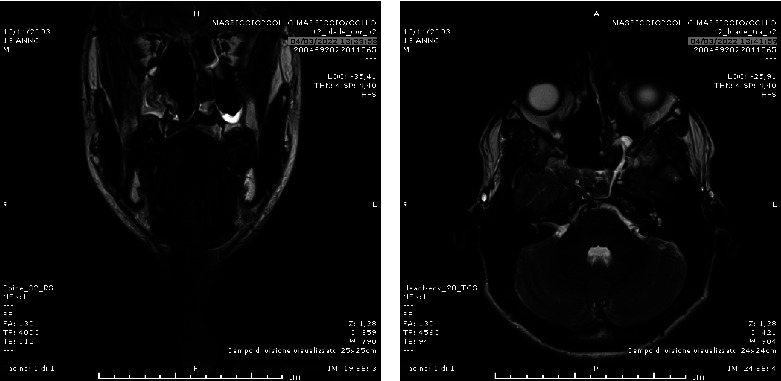
Postprocedural MRI scan performed at 1 month after surgery: coronal (a) and axial (b) T2-weighted views which show the complete resection of the JNA with empty nasal left fossa, with parietal reactive mucous thickness.

The procedure was performed at the interventional radiology angiographic room by two expert IRs. After right groin puncture and a 6F femoral sheath placement, catheterization of the internal and external carotid artery (ICA/ECA) with angiographic study of JNA vascular region was performed for each side. Then, a 6F guide catheter (Envoy MPC 90 cm, Cordis) in proximal ECA and superselective catheterization of lesion feeders was executed (Figure 2(a)) using a balloon-microcatheter (Fr Scepter-C, 0.0165 in, Microvention) and 0.14″ guidewire (Synchro 14, Stryker) for each side. A control run was then performed from the microcatheter to look for dangerous collaterals and determine the precise position of the distal tip. Embolization was then performed using the Onyx 18 liquid embolic system (LES, 6%EVOH, viscosity of 18 cSt, Medtronic) in a slow infusion using blank roadmap visualization to achieve as proper distal penetration as anatomically possible until complete stasis of flow within each feeding vessel was achieved (Figure 2(b)). At the end of the procedure, control angiography was performed to assess the percentage of tumor feeders embolized (Figure 3). Successful embolization was determined as a lack of contrast in the vascular territory of the embolized vessel as showed in Figures 3(b)–3(d). Feeders from the ICA were not treated. The surgeon documented some advantages in the intra and postoperative phases, such as cleaner operating field; better visualization of the lesion thanks to the clear demarcation; easier and faster eradication; better anesthetic management thanks to the parameters maintained in the range of normality. All these vantages corresponded to a shorter operating time (2.40 hours). The blood loss was less than 100 ml. The follow-up MRI performed 1 month after surgical treatment showed an empty nasal fossa in surgical outcomes (Figure 4). The mean hospital stay was 5 days for both embolization and surgery.

## 3. Discussion

Relatively to the rarity of the pathology, currently few works are reported in the literature concerning the treatment of JNA and in any case these regarded limited cases.

By now, the role of preoperative embolization in the JNA treatment has been well-established in terms of safety and efficacy. It guarantees the reduction of blood tumor lesion supply and therefore of intraoperative and postoperative bleeding complications.

There are two kinds of main embolization techniques: intraarterial embolization and the more recent, direct intratumoral embolization.

The most widely used embolic agents are polyvinyl alcohol (PVA) [3], NBCNA (n-Butyl cyanoacrylate), and microparticles/microcoils of different dimensions. However, the embolization with particulate embolic agents is frequently incomplete and time-consuming due to the numerous small arterial feeders, some of which may be inaccessible due to size, tortuosity, or derivation from the internal carotid artery (ICA).

In recent years, for embolization of vascular lesions in the head and neck areas, the use of nonadhesive liquid embolizing agents, such as Onyx, has been spread, especially for arterovenous malformation (AVM) treatment. The advantages of liquid embolic agents are the optimal visualization and the deep penetration and definitive and superselective embolization compared to coils and microparticles/microcoils which are associated also with nontarget embolization. The NBCNA (n-Butyl cyanoacrylate) is more similar to liquid agents, but it has more complex handling [3, 4].

Although Onyx formulation 34 (more viscous) is more frequently used, in the literature also, formulation 18 (more fluid) has been employed [9, 10] for hypervascular lesion treatment and both can be used in the same procedure [6].

The case we describe involves a young patient who presented IV stage JNA which required a selective preoperative endovascular embolization approach. The complexity of the case was related to the size of the tumor lesion and the intracranial extension, which included a vascularization also coming from the ICA. The possible complications related to such embolization were as follows: retrograde reflux and nontarget embolization, which required a careful preoperative angiographic vascular study and a continuous intraoperative evaluation.

The approach chosen to prevent these possible complications has provided for the use of only Onyx 18 with the balloon-assisted technique cuffed in the external carotid artery (ECA).

In the literature, a cuffing technique of the balloon in the ICA is described in order to prevent nontarget embolization in sensitive areas through the possible external internal anastomosis of large tumor lesions [11].

However, in our work, we want to show that also the only selective ECA-cuffed balloon-assisted technique is effective for JNA embolization, avoiding the temporary ICA stop-flow and guaranteeing a safe procedural profile also in patients not fitting for the ICA occlusion test without a significant blood loss.

The novelty of the work consists in the use of a fluid liquid embolic agent (Onyx 18) instead of a particulate agent or viscous liquid (Onyx 34) and in a selective and exclusive ECA branch cuffing.

Differently from Rosenbaum-Halevi et al. [11], which reported successfully a series of 9 cases of JNA embolized with Onyx formulation 34, we used the formulation 18, providing a deeper penetration into tumor capillaries, improving fluoroscopic visibility, and reducing the risk of catheter adherence and secondary vessel injury. Even more, differently from us, they used a double balloon-assisted embolization (BAE) technique, inflating balloons both in ECA and ICA.

The advantage of using the microcatheter with an ECA balloon inflated, immediately upstream of the vascular branches for the tumor lesion, consists in the prevention of retrograde reflux of the embolizing agent and above all in the selectivity of the distribution of the embolizing agent itself. In particular, the Onyx formulation 18, more fluid and less viscous, guarantees a peripheral and capillary distribution inside the tumor [9]. The disadvantage of the uncontrolled distribution of such a fluid agent is overcome thanks to the cuffing of the balloon through the microcatheter because the distribution of the embolizing agent is exclusively dependent on the thrust imparted by the IR and does not depend in any way on the vascular flow, reducing as much as possible the opening of new peripheral anastomoses. However, everything takes place in real time under fluoroscopic guidance in order to have maximum intraoperative control.

Although the presence of an inflated balloon in ICA could reduce the potential migration of embolic material into ICA branches through ECA anastomosis, the selective exclusion of ICA is not always possible, in those patients unable to tolerate ICA sacrifice (in relation to anatomic variants) [12].

Even more, in our opinion, the absence of stop flow in the ICA preserves the blood flow in any ICA-ECA anastomosis, guaranteeing an antagonistic resistance flow to the direction of the embolization injection and avoiding the opening of further anastomotic escape circles at the risk of nontarget embolization.

**Figure 5:**
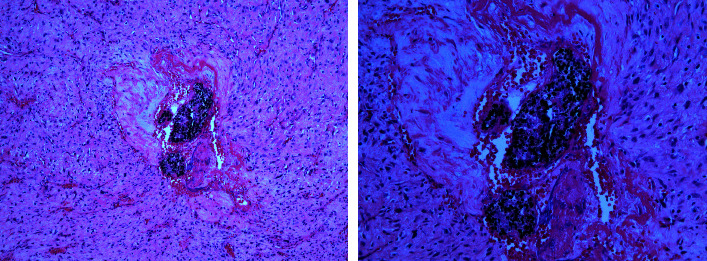
10x magnification of tumor specimen that documents the precise localization with accumulation of the embolizing fluid within the vascular branches and the preserved cytoarchitecture of the tumor demonstrating the selectivity and the optimal distribution and penetration exclusively within the arterial branches.

The advantages and novelty of this technique are as follows: the exclusive occlusion of the afferent branches of the ECA without procedural involvement of the ICA; the possibility of having a modulation, therefore, a control, operator dependent on the injection; an extremely capillary permeation of the embolizing agent, which is also demonstrated on the surgical sample in Figure 5. The preoperative embolization approach used has been reflected in a number of operative advantages, including better visualization and demarcation of the lesion, better control of vital signs, and less intraoperative blood loss (<100 mL). All this led to a faster procedure and shorter hospital stay with positive consequences both for the patient and for his surgical managemen.

## 4. Conclusions

In conclusion, our case showed that the embolization using Onyx 18, with a single stop-flow of the ECA technique, is absolutely definitive and controlled, demonstrating optimal preoperative success in terms of efficacy and safety also in a case of stage IV JNA.

## Data Availability

The data that support the findings of this study are available from the corresponding author upon request.

## Abbreviations

AVM: Artero-venous malformation
DSA: Digital subtraction angiography
ECA: External carotid artery
ICA: Internal carotid artery
IR: Interventional radiologist
JNA: Juvenile nasopharyngeal angiofibroma
MRI: Magnetic resonance imaging
NBCNA: N-butyl cyanoacrylate
PVA: Polyvinyl alcohol.
